# The effectiveness of basivertebral nerve radiofrequency ablation for the treatment of vertebrogenic low back pain: 1-year results of a prospective real-world cohort study

**DOI:** 10.1093/pm/pnaf122

**Published:** 2025-09-02

**Authors:** Zachary L McCormick, Alexandra E Fogarty, Aaron Conger, Taylor Burnham, Richard Kendall, Blake A Dickenson, Napatpaphan Kanjanapanang, Timothy M Curtis, Hasan Sen, Allison Glinka Przybysz, Tyler Clark, Katharine Smolinski, Graham Wagner, Masaru Teramoto, Amanda N Cooper

**Affiliations:** Department of Physical Medicine and Rehabilitation, University of Utah School of Medicine, Salt Lake City, UT 84108, United States; Department of Physical Medicine and Rehabilitation, University of Utah School of Medicine, Salt Lake City, UT 84108, United States; Department of Physical Medicine and Rehabilitation, University of Utah School of Medicine, Salt Lake City, UT 84108, United States; Vivo Cura Health, Calgary, AB, Canada; Department of Physical Medicine and Rehabilitation, University of Utah School of Medicine, Salt Lake City, UT 84108, United States; Department of Physical Medicine and Rehabilitation, University of Utah School of Medicine, Salt Lake City, UT 84108, United States; Department of Physical Medicine and Rehabilitation, University of Utah School of Medicine, Salt Lake City, UT 84108, United States; Department of Physical Medicine and Rehabilitation, University of Utah School of Medicine, Salt Lake City, UT 84108, United States; Department of Physical Medicine and Rehabilitation, University of Utah School of Medicine, Salt Lake City, UT 84108, United States; Department of Physical Medicine and Rehabilitation, University of Utah School of Medicine, Salt Lake City, UT 84108, United States; Department of Physical Medicine and Rehabilitation, University of Utah School of Medicine, Salt Lake City, UT 84108, United States; Department of Physical Medicine and Rehabilitation, University of Utah School of Medicine, Salt Lake City, UT 84108, United States; Department of Physical Medicine and Rehabilitation, University of Utah School of Medicine, Salt Lake City, UT 84108, United States; Department of Physical Medicine and Rehabilitation, University of Utah School of Medicine, Salt Lake City, UT 84108, United States; Department of Physical Medicine and Rehabilitation, University of Utah School of Medicine, Salt Lake City, UT 84108, United States

**Keywords:** low back pain, endplate, chronic, outcomes

## Abstract

**Summary of Background Data:**

Multiple clinical trials have demonstrated the effectiveness of intraosseous basivertebral nerve radiofrequency ablation (BVNA) for treating chronic vertebrogenic low back pain (vLBP). Few studies have evaluated the effectiveness in a real-world population.

**Objectives:**

To evaluate the effectiveness of BVNA for vLBP in a real-world population.

**Methods:**

A single-arm prospective cohort study of patients with LBP ≥ 6 months and Type 1 or Type 2 Modic changes on MRI. The primary outcome was mean improvement in Oswestry Disability Index (ODI) post-BVNA. Secondary outcomes included the proportion of participants with (1) ≥30% and ≥15-point ODI improvements, (2) ≥2-point and ≥50% reductions in pain on Numerical Rating Scale (NRS), and (3) ≥ “much improved” on Patient Global Impression of Change (PGIC) at 3- and 12-month follow-up.

**Results:**

In total, 60 participants were included (mean age 57.0 ± 13.4 years; 45.0% female). Mean ODI score improvement was 13.9 ± 18.7 and 14.0 ± 15.7 points at 3- and 12-month follow-up, respectively. At 12 months, 52.8% (95% CI, 39.7-65.6) of participants reported ≥30% ODI improvement and 39.6% (95% CI, 27.6-53.1) of participants reported ≥15-point improvement in ODI. Twelve-month responder rates for ≥2-point and ≥50% NRS improvement were 67.9% (95% CI, 54.5-78.9) and 49.1% (95% CI, 36.1-62.1). Moreover, 54.7% (95% CI, 41.5-67.3) of participants reported being “much or very much improved” on the PGIC at 12 months.

**Discussion/Conclusion:**

In this real-world cohort, over half of participants with vLBP experienced clinically meaningful improvements in pain and function at 12-month post-BVNA.

**Trial Registration Details:**

ClinicalTrials.gov (original study: NCT04449835; continuation study: NCT05660512); [original study: June 25, 2020; continuation study: December 13, 2022].

## Introduction

Vertebrogenic pain has been established as a specific phenotype of chronic low back pain (LBP) through cumulative basic science, neuroanatomical study, and correlation with a clinical syndrome and imaging biomarkers.^1–6^ The basivertebral nerve provides sensory innervation to the vertebral endplates. As such, basivertebral nerve radiofrequency ablation (BVNA) was introduced as a targeted method of reducing or eliminating chronic vertebrogenic LBP (vLBP). Clinical trials and numerous cohort studies have demonstrated the efficacy and effectiveness of BVNA for well-selected patients with vLBP.^7–12^ Pooled analyses of the randomized trials and prospective cohort studies have demonstrated reductions in healthcare utilization following this therapy,^13^ as well as the cost-effectiveness of BVNA compared to conventional nonsurgical management of vLBP.^14^

A smaller amount of real-world evidence has been reported. In these studies, clinician-investigators reported on cohorts of patients treated with BVNA using less stringent inclusion criteria compared to the original randomized controlled trials and early prospective cohort studies.^15–18^ These studies are limited by their retrospective design,^15–18^ the use of unconventional technology for BVNA that may carry elevated risk,^18^ and/or assessment of a generally older population and one with more comorbidities than what is represented in many typical spine and pain clinics.^15^^,^^16^ While these results hold value for understanding the effectiveness of BVNA in such sub-cohorts, additional study of the more typical vLBP population is needed.

In this context, we aimed to prospectively evaluate outcomes of BVNA in a real-world clinical population with clinically suspected vLBP.

## Methods

### Study design and patient selection

This IRB-approved (IRB_130205), single-group, prospective cohort study was conducted at a single tertiary academic center within the Department of Physical Medicine and Rehabilitation. Participants were recruited and treated between 2020 and 2023. All participants provided written informed consent before undergoing treatment intervention. Coverage for all interventions in this study was provided by participants’ insurance benefits only. No aspect of the BVNA procedures or any other care received was funded by industry. Included were participants who (1) had clinically suspected vLBP based on history, physical examination, and imaging as determined by the enrolling investigator, (2) had Type 1 and/or Type 2 Modic changes on lumbar spine MRI at the levels of planned treatment with BVNA, (3) had failed to respond to at least 6 months of conservative care, (4) agreed to participate in the study, and (5) received BVNA.

### Study intervention

Participants received BVNA treatment at each vertebral level exhibiting Type 1 or Type 2 Modic changes (L2 to S1). The procedure was performed using the Intracept System (Relievant Medsystems, Minneapolis, MN, USA) as described previously with the exception that 7-minute ablation times were used in a subset of participants (vs only 15 minutes in earlier studies).^8^^,^^10^^,^^11^

### Usual care interventions following the study intervention

Usual care post-procedure treatments were provided as deemed appropriate based on a shared decision-making process between the participant and the treating physician, including but not limited to analgesic medications, lumbosacral injections, radiofrequency ablation, and spine surgery. Treatments were categorized for analysis as (1) opioid medication utilization (“opioids”), (2) diagnostic and therapeutic lumbosacral spinal injections (including epidural, facet joint, medial branch, sacroiliac joint, nerve root, trigger point, and discography injections), (3) lumbosacral radiofrequency ablation (LRFA), and (4) lumbosacral spine surgery.

### Data collection

Baseline demographic and clinical information was collected at the time of study enrollment. Baseline variables included age, sex, BMI, duration of LBP, work status, and history of prior lumbosacral spine procedures/surgeries. Validated patient-reported outcome measures (PROMs) were collected at baseline by third party research assistants uninvolved with patient care or study interventions. These included (1) Oswestry Disability Index (ODI) score and (2) typical numerical rating scale (NRS) for LBP score over the past 7 days. LBP-related healthcare utilization data were also collected at baseline, including (1) opioids used for LBP within the past 30 days, (2) injections and LRFAs in the past 12 months, and (3) any prior history of lumbosacral spine surgery.

The same third-party research assistants collected PROMs and LBP-related healthcare utilization data at 3 and 12 months following the study intervention. In addition to the same PROMs collected at baseline, Patient Global Impression of Change (PGIC) scores and work status were collected at each follow-up interval. Follow-up LBP-related healthcare utilization data collection included opioids used for LBP within 30 days of the visit, as well as injections, LRFAs, spine surgeries, and adverse events since the last study visit.

### Data analysis

#### Outcomes

The primary study outcome was the mean improvement in ODI score from baseline to 3 months post-procedure. Continuous secondary outcomes included mean ODI score improvement from baseline at 12 months, mean reduction in NRS LBP from baseline at each follow-up time point, and procedure-related adverse events including new LBP and/or leg pain. Additionally, categorical responder rates were assessed for ODI, NRS, and PGIC at each follow-up period. Categorical secondary outcomes included the respective proportions of participants with ≥15-point and ≥30% improvements in ODI, ≥2-point (the minimal clinically important change [MCIC]^19^) and ≥50% reduction in NRS LBP score, and PGIC score of 6 or 7 (“much improved” or “very much improved”). The proportion of participants categorized as having minimal, moderate, severe, and crippling disability on ODI, as defined by Fairbank and Pynsent,^20^ was also examined at baseline and each follow-up time point as per previous study.^15^

Changes in opioid utilization from baseline were calculated at each follow-up time point as the proportions of participants who had (1) discontinued opioids, (2) continued using opioids, (3) initiated opioids, and (4) remained opioid-free. Additionally, pre- versus post-BVNA opioid utilization was evaluated by comparing the proportions of participants (*n *= 53) who reported using opioid medications at baseline versus 12-month follow-up. For the 53 participants who completed a 12-month follow-up visit, utilization of diagnostic and therapeutic lumbosacral spinal injections, medial/lateral branch LRFA procedures, and lumbosacral spine surgeries post-BVNA was evaluated. Additionally, the proportions of participants who utilized lumbosacral spinal injections and medial/lateral branch LRFAs within the 12 months prior to baseline versus 12 months post-BVNA were compared, as were the total number of interventions (injections and LRFAs) between these time periods. Interventions and surgeries were included in the analysis if they were used for the same pain source and/or location(s) as the participant’s BVNA procedure.

#### Statistical analysis

Descriptive statistics, including frequencies/percentages for categorical variables, and means/standard deviations (SDs) for continuous variables, were calculated for baseline demographic and clinical variables. Furthermore, 95% confidence intervals (CIs) were computed for select statistics. As multivariate analysis, linear regression models with the robust or sandwich estimator of variance^21–23^ were fit to the data on continuous outcome variables measured at 3 months, while using baseline opioid use, age, and baseline score of the corresponding outcome variable as covariates. Regression coefficients (*B*) and their 95% CIs, as well as predicted means, were calculated to aid in interpretations. Dichotomous categorical outcome variables at 3 months were analyzed using Poisson regression models with the robust or sandwich estimator of variance,^21–23^ including the same covariates above, followed by the calculations of incidence rate ratios (IRRs; as measure of prevalence ratio) and their 95% CIs, along with predicted probabilities. Poisson models were chosen over logistic regression models, as recommended by recent literature,^24–26^ since the prevalence ratio is more intuitive and easier to interpret compared with the odds ratio. Longitudinal changes in the outcome variables through 12 months were examined using mixed-effects linear and Poisson regression models^27–30^ for continuous and dichotomous outcome variables, respectively, with the same set of covariates above, along with follow-up time as another covariate. Finally, the proportions of participants utilizing opioids, lumbosacral interventions, and surgeries at 12 months pre- versus post-BVNA were compared using McNemar’s exact test. All analyses were performed using Stata 18.0 (StataCorp LLC, College Station, TX), with an *α* level of .05 for statistical significance.

## Results

A total of 60 participants were treated with BVNA, with 95.0% (*n *= 57) and 88.3% (*n *= 53) going on to complete 3- and 12-month follow-up visits, respectively. All standard and mixed-effects models with results of linear and Poisson regression analyses are included in Tables S1 and S2.

### Baseline demographics and clinical characteristics

Baseline characteristics of participants are summarized in Table 1. Data from a total of 60 participants (*n *= 27 or 45.0% females; 57.0 ± 13.4 years of age; *n *= 54 or 90.0% non-Hispanic/Latino) were analyzed. The majority of participants (*n *= 45; 75.0%) had LBP for ≥5 years, whereas 11.7% (*n *= 7) had LBP for ≥3 years to <5 years. Over a quarter of participants (*n *= 16; 26.7%) reported that LBP had no impact on their ability to work, while 20.0% (*n *= 12) reported working with restrictions. The spinal levels most frequently treated with BVNA were L5 (*n *= 57; 95.0%) and S1 (*n *= 45; 75.0%). The majority of BVNA procedures utilized 15-minute ablations at all levels of treatment (*n *= 34; 56.7%), with 7-minute ablations used exclusively in only 18.3% (*n *= 11) of procedures.

**Table 1.** Baseline demographics and clinical characteristics of participants (*N *= 60).

Variable	No. (%)
Gender	
Male	33 (55.0)
Female	27 (45.0)
Ethnicity	
Not Hispanic/Latino	54 (90.0)
Hispanic/Latino	6 (10.0)
Procedure funding source	
Private insurance	34 (56.7)
Medicare	23 (38.3)
Tricare	1 (1.7)
Other	2 (3.3)
Low back pain duration	
≥6 months to <1 year	2 (3.3)
≥1 year to <3 years	6 (10.0)
≥3 years to <5 years	7 (11.7)
≥5 years	45 (75.0)
Work status related to low back pain	
No impact to ability to work	16 (26.7)
Working with restrictions	12 (20.0)
Had to change jobs due to low back pain	2 (3.3)
Unable to work at all/disabled due to low back pain	2 (3.3)
Not currently working, unrelated to low back pain	25 (41.7)
Other	3 (5.0)
Opioid medication(s) prescribed for low back pain	
Yes	22 (36.7)
No	38 (63.3)
Opioid medication(s) utilized in the past 30 days	
Yes	20 (33.3)
No	40 (66.7)
Lumbosacral injection utilization in the past 12 months	
Yes	41 (68.3)
No	19 (31.7)
Lumbosacral medial/lateral branch RFA in the past 12 months	
Yes	14 (23.3)
No	46 (76.7)
History of lumbar surgery	
Non-fusion surgery	7 (11.7)
Fusion surgery	1 (1.7)
Levels treated by BVNA	
L1	0 (0.0)
L2	6 (10.0)
L3	19 (31.7)
L4	34 (56.7)
L5	57 (95.0)
S1	45 (75.0)
BVNA ablation time	
15 minutes for all ablations	34 (56.7)
7 minutes for all ablations	11 (18.3)
Both 15- and 7-minute ablations used	15 (25.0)
Age (mean ± SD)	57.0 ± 13.4
Baseline ODI (mean ± SD)	38.7 ± 13.9
Baseline NRS (mean ± SD)	6.3 ± 1.9

Missing values are excluded. BVNA, basivertebral nerve ablation; NRS, Numerical Rating Scale; ODI, Oswestry Disability Index; RFA, radiofrequency ablation; SD, standard deviation.

### Oswestry Disability Index

Continuous ODI score reductions from baseline, along with categorical outcomes for ≥15-point and ≥30% ODI reduction, are presented for each follow-up time point in Table 2. ODI categories at baseline and at each follow-up time point are shown in Table 3. At 3 months, the average ODI score was reduced from baseline by 13.9 ± 18.7 points. At 12 months, the mean overall ODI score reduction was 14.0 ± 15.7 points from baseline. Mixed-effects linear regression analysis showed baseline opioid use was significantly associated with smaller reductions in ODI score by approximately 9 points when adjusting for age, baseline ODI score, and follow-up time (*B *= −8.77; 95% CI, −15.30, −2.24; *P *= .008; Table S1b).

**Table 2.** Oswestry Disability Index reductions from baseline at 3 and 12 months after basivertebral nerve radiofrequency ablation.

Outcome variable	3 months	12 months
	(*N *= 57)	(*N *= 53)
ODI score reduction (mean ± SD)	13.9 ± 18.7	14.0 ± 15.7
≥15-point ODI reduction		
Yes	27 (47.4)	21 (39.6)
No	30 (52.6)	32 (60.4)
≥30% ODI reduction		
Yes	35 (61.4)	28 (52.8)
No	22 (38.6)	25 (47.2)

Values are frequency (%), unless specified otherwise. ODI, Oswestry Disability Index; SD, standard deviation.

**Table 3.** Oswestry Disability Index categories at baseline and follow-up time points.

ODI category	Baseline	3 months	12 months
	(*N *= 60)	(*N *= 57)	(*N *= 53)
Minimal disability	4 (6.7) 2.6, 15.9	27 (47.4) 35.0, 60.1	24 (45.3) 32.7, 58.6
Moderate disability	33 (55.0) 42.5, 66.9	22 (38.6) 27.1, 51.6	23 (43.4) 31.0, 56.7
Severe disability	19 (31.7) 21.3, 44.2	7 (12.3) 6.1, 23.3	6 (11.3) 5.3, 22.6
Crippled	4 (6.7) 2.6, 15.9	1 (1.8) 0.3, 9.3	0 (0.0) 0.0, 6.8

Values are frequency (%) with 95% confidence intervals. ODI, Oswestry Disability Index.

At 3 months, 47.4% (*n *= 27; 95% CI, 35.0, 60.1) and 61.4% (*n *= 35; 95% CI, 48.4, 72.9) of participants reported ≥15-point and ≥30% ODI reductions, respectively. At 12 months, 39.6% (*n *= 21; 95% CI, 27.6, 53.1) and 52.8% (*n *= 28; 95% CI, 39.7, 65.6) of participants reported ≥15-point and ≥30% ODI reductions, respectively.

### Numerical rating scale

Continuous NRS score reductions from baseline, along with categorical outcomes for ≥2-point and ≥50% NRS reduction, are summarized by follow-up time in Table 4. At 3 months, the mean NRS score reduction from baseline was 2.4 ± 2.7 points. At 12 months, the mean NRS score reduction from baseline was 2.8 ± 2.8 points. After adjusting for opioid use, baseline NRS score, and follow-up time, age demonstrated a slight negative, yet significant association with NRS score reduction (*B *= −0.04; 95% CI, −0.08, −0.01; *P *= .015; Table S1b).

**Table 4.** Pain score reductions from baseline at 3 and 12 months after basivertebral nerve radiofrequency ablation.

Outcome variable	3 months	12 months
	(*N *= 57)	(*N *= 53)
NRS score reduction (mean ± SD)	2.4 ± 2.7	2.8 ± 2.8
≥2-point NRS reduction		
Yes	33 (57.9)	36 (67.9)
No	24 (42.1)	17 (32.1)
≥50% NRS reduction		
Yes	24 (42.1)	26 (49.1)
No	33 (57.9)	27 (50.9)

Values are frequency (%), unless specified otherwise. NRS, Numerical Rating Scale; SD, standard deviation.

Overall, 57.9% (*n *= 33; 95% CI, 45.0, 69.8) and 42.1% (*n *= 24; 95% CI, 30.2, 55.0) of participants demonstrated ≥2-point and ≥50% NRS reductions at 3 months, respectively. Twelve-month responder rates for ≥2-point and ≥50% NRS reductions were 67.9% (*n *= 36; 95% CI, 54.5, 78.9) and 49.1% (*n *= 26; 95% CI, 36.1, 62.1).

### Patient global impression of change

Outcomes for PGIC are presented for each follow-up time point in Table 5. At 3 months, 54.4% (*n *= 31; 95% CI, 41.6, 66.6) of participants reported PGIC scores ≥6, consistent with “much improved” or “very much improved.” Twelve-month responder rates for PGIC were 54.7% (*n *= 29; 95% CI, 41.5, 67.3). The mixed-effects Poisson regression model for ≥6 on PGIC revealed a negative, significant association with age, where the likelihood of achieving this outcome decreased by 2% for each additional year after adjusting for baseline opioid use and follow-up time (IRR = 0.98; 95% CI, 0.97, 0.99; *P *= .021; Table S2b).

**Table 5.** Patient global impression of change at 3 and 12 months after basivertebral nerve radiofrequency ablation.

≥6 PGIC	3 months	12 months
	(*N *= 57)	(*N *= 53)
Yes	31 (54.4)	29 (54.7)
No	26 (45.6)	24 (45.3)

PGIC scores ≥6 indicate “much improved” or “very much improved.” PGIC, Patient Global Impression of Change.

### Impact of LBP on ability to work and activity level

Self-reported impact of LBP on ability to work at baseline and each follow-up interval is shown in Table S3. Self-reported activity level at each follow-up interval is shown in Table S4.

### LBP-related medication and healthcare utilization at 12 months pre- versus post-BVNA

Changes in participant-reported opioid utilization from baseline to 3 and 12 months post-BVNA are summarized in Table 6, while comparisons of medication and procedural utilization at 12 months pre- versus post-BVNA are presented in Table 7 for the 53 participants who completed a 12-month follow-up visit. Twelve-month responder rates for ODI, NRS, and PGIC are stratified by medication and procedural utilization status in Tables S5-S7.

**Table 6.** Changes to opioid utilization at 3 and 12 months after basivertebral nerve radiofrequency ablation compared to baseline.

Opioid usage	3 months	12 months
	(*N *= 57)	(*N *= 53)
Yes (baseline) → No	8 (14.0)	6 (11.3)
Yes (baseline) → Yes	11 (19.3)	11 (20.8)
No (baseline) → Yes	5 (8.8)	7 (13.2)
No (baseline) → No	33 (57.9)	29 (54.7)

**Table 7.** Participants utilizing opioids, lumbosacral interventions, and surgeries at 12 months pre- and post-basivertebral nerve radiofrequency ablation (*N *= 53 for this analysis).

Treatment type	12 months prior to baseline	12 months post-BVNA	P*	Difference in proportions (95% CI)
Opioids at baseline vs 12 months^a^	17 (32.1)	18 (34.0)	.999	1.9 (-17.1, 13.3)
Of those using opioids at baseline^b^	17 (100.0)	11 (64.7)	**.031**	35.3 (6.7, 63.9)
Injections	36 (67.9)	11 (20.8)	**<.001**	47.2 (29.1, 65.3)
Epidural	21 (39.6)	4 (7.5)	**<.001**	32.1 (15.6, 48.5)
Facet block/injection	6 (11.3)	0 (0.0)	**.031**	11.3 (0.9, 21.7)
Medial branch block/injection	3 (5.7)	2 (3.8)	.999	1.9 (-8.2, 12.0)
SI joint	1 (1.9)	1 (1.9)	.999	0.0 (-7.1, 7.1)
Nerve root block/injection	0 (0.0)	0 (0.0)	.999	0.0 (-1.9, 1.9)
Trigger point	0 (0.0)	0 (0.0)	.999	0.0 (-1.9, 1.9)
Discography	5 (9.4)	0 (0.0)	.063	9.4 (-0.3, 19.2)
Other	5 (9.4)	4 (7.5)	.999	1.9 (-11.1, 14.9)
Medial/lateral branch LRFAs	13 (24.5)	5 (9.4)	**.039**	15.1 (1.1, 29.1)
Surgeries	0 (0.0)	3 (5.7)	.250	5.7 (-13.8,2.4)
Discectomy	0 (0.0)	0 (0.0)	.999	0.0 (-1.9, 1.9)
Laminectomy/laminotomy	0 (0.0)	1 (1.9)	.999	1.9 (-7.4, 3.7)
Foraminotomy	0 (0.0)	1 (1.9)	.999	1.9 (-7.4, 3.7)
Fusion	0 (0.0)	1 (1.9)	.999	1.9 (-7.4, 3.7)
Total disc replacement	0 (0.0)	0 (0.0)	.999	0.0 (-1.9, 1.9)
Neurostimulator	0 (0.0)	0 (0.0)	.999	0.0 (-1.9, 1.9)
Other	0 (0.0)	0 (0.0)	.999	0.0 (-1.9, 1.9)

Values are frequency (%), unless specified otherwise. BVNA, basivertebral nerve ablation; CI, confidence interval; LRFA, lumbosacral radiofrequency ablation; SI, sacroiliac.

* From McNemar’s exact test. Statistically significant values are shown in bold.

a Defined as utilization of opioid medication(s) for low back pain in the past 30 days prior to study visit.

b Calculations for *n *= 17 participants using opioids at baseline.

Of the 17 participants consuming opioid analgesics at baseline, 6 participants (35.3%; 95% CI, 6.7, 63.9; *P *= .031; Table 7) had ceased opioid use at 12 months post-BVNA. Alternatively, among participants not consuming opioids at baseline, 7 of 36 participants (19.4%; 95% CI, 3.7, 35.2; *P *= .016) had started consuming opioids at 12 months post-BVNA.

Significant decreases were observed in utilization of lumbosacral interventions performed in the 12 months pre- versus post-BVNA. In the 12-month period prior to baseline, 36 participants received a total of 56 injections compared to 11 participants who received 14 injections at 12 months post-BVNA, constituting a 47.2% decrease in the proportion of participants receiving injections (95% CI, 29.1, 65.3; *P *< .001). Regarding specific types of injections, significant decreases from pre- to post-BVNA were apparent for epidural (21 vs 4 participants; 32.1%; 95% CI, 15.6, 48.5; *P *< .001) and facet joint injections (6 vs 0 participants; 11.3%; 95% CI, 0.9, 21.7; *P *= .031). Compared to the 14 medial/lateral branch LRFAs performed for 13 (24.5%) participants in the 12 months prior to baseline assessment, 5 (9.4%) participants had a total of 6 LRFA procedures post-BVNA, representing a statistically significant reduction of 15.1% (95% CI, 1.1, 29.1; *P *= .039). Three (5.7%) participants underwent a lumbosacral surgical procedure in the 12 months post-BVNA (1 laminectomy, 1 foraminotomy, and 1 fusion surgery, representing 1.9% of 53 participants each).

### BVNA duration

An additional, exploratory sub-analysis was performed by adding BVNA time (Table 1) as a covariate to the regression models described above for all study outcomes, in order to test for disparities between BVNA ablation protocols (15-minute ablations at all treatment levels versus at least one 7-minute ablation). The analysis revealed no significant differences in outcomes between participants treated exclusively with 15-minute ablation durations at all levels treated compared to those with 7-minute ablation times one or more levels treated.

### Adverse events

All adverse events (AEs) were captured and assessed throughout the study period, regardless of their associations with the procedure or device. Each AE was adjudicated by the principal investigator (ZM), who determined the severity and relationship to the study intervention.

A total of 13 AEs were reported for 9 participants. Nine AEs ranging from mild to moderate severity were adjudicated as unrelated to the procedure. These included a fall that required no treatment and was associated with no sequelae, 2 falls that required shoulder surgery but were associated with no ongoing sequelae, hip pain from prior labral repair surgery, return of prior radicular pain and intermittent sciatica/numbness, reported >3 months post-BVNA procedure, new buttock pain treated with oral medications, L4-L5 disc re-herniation with pain resolving after an epidural steroid injection, and worsening spine pain with radicular symptoms due to central canal stenosis that resolved with decompression surgery.

Four AEs were procedure-related, including one serious adverse event (SAE) in a patient who experienced intense pain along the left buttock, lateral thigh, and calf within hours following the procedure. Subsequent MRI/CT imaging revealed pedicle breach with a small bone fragment in proximity to the L5 nerve root, leading to a diagnosis of L5 radiculopathy with foot drop. No further surgical intervention was recommended due to absence of ongoing nerve compression. Although improvement was noted with transforaminal epidural steroid injections, physical therapy, and oral medications, the issue remained unresolved at follow-up as the patient still experienced mild, ongoing residual weakness without leg pain at 12 months. The 3 remaining procedure-related AEs were mild, comprising an inaccessible treatment level (S1) during the procedure, hip dysesthesia that resolved without further treatment, and leg numbness/weakness that resolved with physical therapy.

## Discussion

Chronic LBP continues to pose a major challenge for patients and clinicians alike, often leading to long-term disability, diminished quality of life, and high healthcare costs. Among the various etiologies of chronic LBP, vertebrogenic pain has emerged as a distinct and increasingly recognized phenotype. This prospective cohort study provides real-world evidence on the effectiveness of BVNA in a diverse population treated within routine clinical practice, offering insights beyond those captured in controlled trial settings. Notably, participants in this study sample included individuals with a history of spinal fusion and/or decompressive surgery. Individuals with a history of radicular pain and/or neurogenic claudication in the context of radiographic stenosis, but who had responded to treatment of the neuropathic component of their pain prior to being considered for BVNA (ie, their remaining pain was axial-dominant), were also included. Individuals in this sample may have also had concomitant non-vertebrogenic pain generators (ie, facet joint, sacroiliac joints, etc), but the enrolling investigator judged the pain to be primarily vertebrogenic and otherwise fit the study inclusion criteria.

Within this real-world study sample, we observed clinically meaningful improvements in both pain and function 3 months post-BVNA, with benefits largely sustained at 12 months. The mean improvement in Oswestry Disability Index (ODI) was 13.9 ± 18.7 at 3 months and 14.0 ± 15.7 at 12 months. At 12-month follow-up, 52.8% (95% CI, 39.7−65.6) of participants reported a  ≥30% improvement in ODI, whereas 39.6% (95% CI, 27.6−53.1) achieved a  ≥15-point absolute reduction. Both thresholds are recognized as MCICs for ODI—a  ≥10- to 15-point absolute reduction or a  ≥30% relative improvement are commonly accepted thresholds.^31^ The discrepancy between percentage-based and absolute responder rates in our cohort likely reflects the relatively modest baseline ODI, as percentage improvements are more sensitive in patients with lower initial disability. Similar trends were observed in pain outcomes: 67.9% (95% CI, 54.5−78.9) of participants achieved a  ≥2-point reduction in numeric rating scale (NRS) score, which meets the MCIC for pain, while 49.1% (95% CI, 36.1−62.1) achieved a  ≥50% reduction-considered a robust clinically significant improvement.^19^^,^^32^ Compared to outcomes from industry-sponsored trials,^9^^,^^10^^,^^12^ these response rates are slightly lower, which is expected in real-world populations. Nevertheless, our findings support the clinical utility of BVNA in broader patient populations.

When comparing to other real-world cohorts, De Vivo et al reported an exceptionally high responder rate for a prospective uncontrolled trial of 56 participants using a tumor ablation system, with 96.5% of patients achieving clinically significant improvements in both visual analog scale (VAS) (≥2 cm) and ODI (≥10 points) at 3 and 12 months. Similarly, in a cohort study of 19 patients using a tumor ablation system, 84.2% of patients undergoing BVNA experienced more than 50% pain reduction across follow-up time points.^17^ However, the use of an off-label device system limits generalizability of these results. Schnapp et al shared results of a study which included an older population (mean age 73 years), reporting that 67.7% of patients experienced ≥15-point ODI improvement and 77.4% had ≥2 cm VAS reductions by 12 months, with all outcomes (ODI, VAS, SF-36 PCS/MCS) showing statistically and clinically significant improvement (*P *< .001), except SF-36 MCS at 1 month (*P *= .165).^15^ A retrospective study by Fogel et al focused on a medically complex cohort with advanced comorbidities including scoliosis and prior laminectomy.^16^ At last follow-up (timeline not standardized), Group A (fewer comorbidities) showed a 7 cm VAS and 39-point ODI improvement, while Group B (more comorbidities) showed a 6 cm VAS and 28-point ODI improvement, though many remained in the moderately disabled range. Compared to these, our findings reflect clinical benefit with BVNA at 12 months in a more representative outpatient spine clinic population.

Regression models used in the present analysis identified baseline opioid use and older age as significant predictors of poorer outcomes post-BVNA. This finding partially aligns with a prior pooled analysis of data from 3 clinical trials, demonstrating that baseline opioid use decreased the odds of ≥15-point ODI reduction after BVNA, albeit with limited predictive capacity.^4^ Alternatively, age was not found to be a significant predictor of treatment success in the pooled analysis from these clinical trials. We suspect that this reflects the more stringent selection criteria within the clinical trials compared to the present real-world study. Older individuals are more likely to have facet joint pain, sacroiliac joint pain, and radiographic evidence of spinal stenosis (with or without symptoms) compared to younger individuals.^33^^,^^34^ Our study sample may have contained more individuals with concomitant non-vertebrogenic pain generators than the clinical trials given our more generous inclusion criteria. Further investigation is warranted to evaluate demographic and clinical factors associated with BVNA treatment response in real-world populations.

Our study demonstrated a meaningful reduction in LBP-related healthcare utilization in the 12 months following BVNA. Most notably, only one participant (1.9%) underwent lumbar fusion during the 12-month follow-up. For context, Mirza et al reported a 14% lumbar fusion rate within just 2.4 months in a similarly defined chronic axial LBP population presenting to a spine clinic, with 17% going on to receive some form of lumbar surgery within 6 months.^35^ Our findings align more closely with a pooled analysis of utilization data from 3 prospective BVNA trials that described lumbar fusion rates of 0.8% at 1 year and 6.5% at a mean follow-up of 5.3 years.^13^ These comparisons highlight a potential long-term surgical-sparing effect of BVNA. In addition, our study showed significant reductions in percutaneous spinal interventions. The proportion of participants receiving lumbosacral spinal injections decreased by 47.2% (from 36 to 11 participants; 95% CI, 29.1−65.3; *P* < .001), with notable decreases in both epidural (21 to 4 participants; *P *< .001) and facet joint injections (6 to 0 participants; *P *= .031). Similarly, LRFA procedures were reduced from 24.5% to 9.4% of participants (*P *= .039). These results mirror findings of an 81.2% reduction in spinal injections at 1 year and sustained reductions over 5 years (76.4%) from prior study.^13^

Opioid utilization following BVNA in our cohort demonstrated minimal overall change, with 32.1% of participants using opioids at baseline and 34.0% at 12 months. At 3 months, 28.1% reported opioid use, and 14.0% had discontinued use since baseline. At 12 months, opioid utilization was reduced by 35.3% among those who reported using opioids at baseline, which is comparable to the 40.3% reduction observed at 1 year in prior study.^13^ However, this reduction was balanced by new utilization of opioids by participants who were not taking them at baseline.

Finally, the majority of BVNA procedures in our study utilized 15-minute ablations at all treatment levels (*n *= 34; 56.7%). While our study was not powered to detect differences by ablation duration, exploratory analysis showed no clear difference in outcomes between 7- and 15-minute ablations. Ablation time at each treatment level was independently decided upon by the treating physician based on his or her perceived targeting of the basivertebral nerve on fluoroscopy. Typically, 7-minute ablations are only utilized when the positioning of the RF probe is expected to result in comprehensive capture of the targeted nerve. In the present study, however, the basivertebral nerve may have been missed or only partially captured in cases where a 7-minute ablation was applied imprecisely. It is possible that using 15-minute ablations exclusively at all treatment levels may have resulted in higher treatment responder rates in our cohort, closer to those observed in the original randomized controlled trials for BVNA.^8–12^

### Limitations

This study has several limitations. It reflects a single-center experience, which may limit the generalizability of the findings to other clinical settings. Given that we did not require diagnostic medial branch blocks or sacroiliac joint injections to rule out pain from these sources prior to study enrollment, it is possible that our cohort included patients without dominant vertebrogenic pain. Outcomes may have been improved if negative responses to diagnostic injections were part of our study eligibility criteria. Although we prospectively collected detailed data on procedural interventions, medication use, and surgical outcomes, we did not capture the full scope of LBP-related healthcare utilization, such as physical therapy, outpatient visits, emergency department utilization, or imaging studies. Finally, the observed reductions in LBP-related healthcare utilization post-BVNA may reflect procedural success and/or a lack of additional treatment options, as many participants in this cohort also failed to respond to interventional pain procedures in addition to conservative care. Further study with a control group is necessary to determine whether BVNA directly decreases utilization of further LBP-related care.

## Conclusion

This prospective, single-center cohort study provides real-world evidence on the clinical effectiveness and healthcare utilization impact of BVNA in patients with chronic vLBP. We observed clinically meaningful improvements in pain and function at 3 months, which were largely sustained through 12 months. The procedure was associated with substantial reductions in spinal injections and radiofrequency ablations, and a very low rate of lumbar fusion at 12 months, supporting its value in reducing LBP-related interventional burden. Our findings support the role of BVNA as an effective treatment option in routine clinical practice, while also underscoring the need for continued research in diverse patient populations.

## Supplementary Material

pnaf122_Supplementary_Data
