# Supplementary material for: The effectiveness of basivertebral nerve radiofrequency ablation for the treatment of vertebrogenic low back pain: 1-year results of a prospective real-world cohort study
Source: Pain Med. 2025 Sep 2;27(3):254–61. doi: 10.1093/pm/pnaf122 (PMC13017880; doi:10.1093/pm/pnaf122)
Supplement: pnaf122_Supplementary_Data [file pnaf122_supplementary_data.zip › SASI Supplemental Tables_updated_15AUG2025.docx]

**Supplemental Table S1a.** Linear regression models on Oswestry Disability Index and pain score reductions with select covariates at 3 months after basivertebral nerve radiofrequency ablation

| **Outcome** | **Covariate** | ***B*** | **95% CI** | ***p*** |
| --- | --- | --- | --- | --- |
| ODI score reduction | Baseline opioid use (vs. no) |  |  |  |
|  | Yes | -7.06 | -16.12, 2.00 | 0.124 |
|  | Age | -0.26 | -0.55, 0.04 | 0.085 |
|  | Baseline ODI score | 0.82 | 0.48, 1.16 | **< 0.001** |
| NRS score reduction | Baseline opioid use (vs. no) |  |  |  |
|  | Yes | -0.50 | -1.83, 0.83 | 0.458 |
|  | Age | -0.05 | -0.09, 0.00 | 0.056 |
|  | Baseline NRS score | 0.63 | 0.32, 0.94 | **< 0.001** |

*B* = beta coefficient; CI = confidence interval; NRS = Numerical Rating Scale; ODI = Oswestry Disability Index.

**Supplemental Table S1b.** Mixed-effects linear regression models on Oswestry Disability Index and pain score reductions with select covariates

| **Outcome** | **Covariate** | ***B*** | **95% CI** | ***p*** |
| --- | --- | --- | --- | --- |
| ODI score reduction | Follow-up (vs. 3 months) |  |  |  |
|  | 12 months | -0.13 | -4.08, 3.82 | 0.949 |
|  | Baseline opioid use (vs. no) |  |  |  |
|  | Yes | -8.77 | -15.30, -2.24 | **0.008** |
|  | Age | -0.20 | -0.41, 0.01 | 0.059 |
|  | Baseline ODI score | 0.77 | 0.53, 1.01 | **< 0.001** |
| NRS score reduction | Follow-up (vs. 3 months) |  |  |  |
|  | 12 months | 0.42 | -0.25, 1.09 | 0.215 |
|  | Baseline opioid use (vs. no) |  |  |  |
|  | Yes | -0.85 | -1.80, 0.10 | 0.081 |
|  | Age | -0.04 | -0.08, -0.01 | **0.015** |
|  | Baseline NRS score | 0.81 | 0.58, 1.03 | **< 0.001** |

*B* = beta coefficient; CI = confidence interval; NRS = Numerical Rating Scale; ODI = Oswestry Disability Index.

Note: An insignificant covariate of ‘follow-up’ indicates no significant differences in outcomes between 3- and 12-month follow-up.

**Supplemental Table S2a.** Poisson models on categorical study outcomes with select covariates at 3 months after basivertebral nerve radiofrequency ablation

| **Outcome** | **Covariate** | **IRR** | **95% CI** | ***p*** |
| --- | --- | --- | --- | --- |
| ≥ 15-point ODI reduction | Baseline opioid use (vs. no) |  |  |  |
|  | Yes | 0.86 | 0.52, 1.43 | 0.559 |
|  | Age | 0.99 | 0.97, 1.01 | 0.436 |
|  | Baseline ODI score | 1.03 | 1.02, 1.05 | **< 0.001** |
| ≥ 30% ODI reduction | Baseline opioid use (vs. no) |  |  |  |
|  | Yes | 0.86 | 0.56, 1.34 | 0.506 |
|  | Age | 0.99 | 0.98, 1.01 | 0.313 |
|  | Baseline ODI score | 1.02 | 1.00, 1.03 | **0.007** |
| ≥ 2-point NRS reduction | Baseline opioid use (vs. no) |  |  |  |
|  | Yes | 0.88 | 0.55, 1.40 | 0.589 |
|  | Age | 0.99 | 0.97, 1.01 | 0.202 |
|  | Baseline NRS score | 1.17 | 1.04, 1.31 | **0.008** |
| ≥ 50% NRS reduction | Baseline opioid use (vs. no) |  |  |  |
|  | Yes | 0.85 | 0.43, 1.69 | 0.652 |
|  | Age | 0.98 | 0.96, 1.01 | 0.145 |
|  | Baseline NRS score | 1.02 | 0.88, 1.19 | 0.778 |
| ≥ 6 PGIC | Baseline opioid use (vs. no) |  |  |  |
|  | Yes | 0.85 | 0.49, 1.45 | 0.541 |
|  | Age | 0.99 | 0.97, 1.00 | 0.105 |

CI = confidence interval; IRR = incidence rate ratio; NRS = Numerical Rating Scale; ODI = Oswestry Disability Index; PGIC = Patient Global Impression of Change.

**Supplemental Table S2b.** Mixed-effects Poisson regression models on categorical study outcomes with select covariates

| **Outcome** | **Covariate** | **IRR** | **95% CI** | ***p*** |
| --- | --- | --- | --- | --- |
| ≥ 15-point ODI reduction | Follow-up (vs. 3 months) |  |  |  |
|  | 12 months | 0.83 | 0.60, 1.15 | 0.270 |
|  | Baseline opioid use (vs. no) |  |  |  |
|  | Yes | 0.65 | 0.41, 1.03 | 0.069 |
|  | Age | 0.99 | 0.97, 1.01 | 0.348 |
|  | Baseline ODI score | 1.04 | 1.02, 1.05 | **< 0.001** |
| ≥ 30% ODI reduction | Follow-up (vs. 3 months) |  |  |  |
|  | 12 months | 0.86 | 0.67, 1.10 | 0.224 |
|  | Baseline opioid use (vs. no) |  |  |  |
|  | Yes | 0.66 | 0.43, 1.02 | 0.059 |
|  | Age | 0.99 | 0.98, 1.00 | 0.165 |
|  | Baseline ODI score | 1.02 | 1.00, 1.03 | **0.004** |
| ≥ 2-point NRS reduction | Follow-up (vs. 3 months) |  |  |  |
|  | 12 months | 1.18 | 0.93, 1.50 | 0.181 |
|  | Baseline opioid use (vs. no) |  |  |  |
|  | Yes | 0.85 | 0.62, 1.18 | 0.337 |
|  | Age | 0.99 | 0.98, 1.00 | 0.180 |
|  | Baseline NRS score | 1.19 | 1.09, 1.31 | **< 0.001** |
| ≥ 50% NRS reduction | Follow-up (vs. 3 months) |  |  |  |
|  | 12 months | 1.17 | 0.84, 1.61 | 0.353 |
|  | Baseline opioid use (vs. no) |  |  |  |
|  | Yes | 0.67 | 0.38, 1.18 | 0.163 |
|  | Age | 0.99 | 0.97, 1.01 | 0.207 |
|  | Baseline NRS score | 1.05 | 0.93, 1.18 | 0.444 |
| ≥ 6 PGIC | Follow-up (vs. 3 months) |  |  |  |
|  | 12 months | 1.01 | 0.81, 1.26 | 0.949 |
|  | Baseline opioid use (vs. no) |  |  |  |
|  | Yes | 0.66 | 0.40, 1.11 | 0.117 |
|  | Age | 0.98 | 0.97, 1.00 | **0.021** |

CI = confidence interval; IRR = incidence rate ratio; NRS = Numerical Rating Scale; ODI = Oswestry Disability Index; PGIC = Patient Global Impression of Change.

Note: An insignificant covariate of ‘follow-up’ indicates no significant differences in outcomes between 3- and 12-month follow-up.

**Supplemental Table S3.** Work status related to low back pain at baseline and follow-up time points

| **Work status** | **Baseline**  **(*N* = 60)** | **3 months**  **(*N* = 57)** | **12 months**  **(*N* = 53)** |
| --- | --- | --- | --- |
| No impact to ability to work | 16 (26.7) | 20 (35.1) | 18 (34.0) |
| Working with restrictions | 12 (20.0) | 8 (14.0) | 3 (5.7) |
| Had to change jobs due to low back pain | 2 (3.3) | 0 (0.0) | 0 (0.0) |
| Unable to work at all/disabled due to low back pain | 2 (3.3) | 4 (7.0) | 4 (7.5) |
| Not currently working, unrelated to low back pain | 25 (41.7) | 23 (40.4) | 23 (43.4) |
| Other | 3 (5.0) | 2 (3.5) | 5 (9.4) |

Note: Values are frequency (%).

**Supplemental Table S4.** Self-reported ability to resume activity level from prior to onset of low back pain at 3 and 12 months after basivertebral nerve radiofrequency ablation

| **Activity level** | **3 months**  **(*N* = 57)** | **12 months**  **(*N* = 53)** |
| --- | --- | --- |
| Yes | 19 (33.3) | 31 (58.5) |
| No | 38 (66.7) | 22 (41.5) |

Note: Values are frequency (%).

**Supplemental Table S5.** Categorical study outcomes by changes to opioid utilization at 12 months after basivertebral nerve ablation compared to baseline

|  | **Changes in Opioid Use from Baseline to 12 Months** | | | |
| --- | --- | --- | --- | --- |
| **Outcome** | **Yes (baseline) -> No**  **(*n* = 6)** | **No (baseline) -> No**  **(*n* = 29)** | **No (baseline) -> Yes**  **(*n* = 7)** | **Yes (baseline) -> Yes**  **(*n* = 11)** |
| ≥ 15-point ODI reduction |  |  |  |  |
| Yes | 2 (33.3) | 14 (48.3) | 3 (42.9) | 2 (18.2) |
| No | 4 (66.7) | 15 (51.7) | 4 (57.1) | 9 (81.8) |
| ≥ 30% ODI reduction |  |  |  |  |
| Yes | 2 (33.3) | 19 (65.5) | 4 (57.1) | 3 (27.3) |
| No | 4 (66.7) | 10 (34.5) | 3 (42.9) | 8 (72.7) |
| ≥ 50% NRS reduction |  |  |  |  |
| Yes | 1 (16.7) | 18 (62.1) | 3 (42.9) | 4 (36.4) |
| No | 5 (83.3) | 11 (37.9) | 4 (57.1) | 7 (63.6) |
| ≥ 2-point NRS reduction |  |  |  |  |
| Yes | 5 (83.3) | 22 (75.9) | 4 (57.1) | 5 (45.5) |
| No | 1 (16.7) | 7 (24.1) | 3 (42.9) | 6 (54.5) |
| ≥ 6 PGIC |  |  |  |  |
| Yes | 0 (0.0) | 20 (69.0) | 4 (57.1) | 5 (45.5) |
| No | 6 (100.0) | 9 (31.0) | 3 (42.9) | 6 (54.5) |

NRS = Numerical Rating Scale; ODI = Oswestry Disability Index; PGIC = Patient Global Impression of Change.

Note: Values are frequency (%).

**Supplemental Table S6.** Categorical study outcomes by changes to injection utilization at 12 months after basivertebral nerve ablation compared to baseline

|  | **Changes in Injection Use from Baseline to 12 Months** | | | |
| --- | --- | --- | --- | --- |
| **Outcome** | **Yes (baseline) -> No**  **(*n* = 29)** | **No (baseline) -> No**  **(*n* = 13)** | **No (baseline) -> Yes**  **(*n* = 4)** | **Yes (baseline) -> Yes**  **(*n* = 7)** |
| ≥ 15-point ODI reduction |  |  |  |  |
| Yes | 15 (51.7) | 3 (23.1) | 1 (25.0) | 2 (28.6) |
| No | 14 (48.3) | 10 (76.9) | 3 (75.0) | 5 (71.4) |
| ≥ 30% ODI reduction |  |  |  |  |
| Yes | 18 (62.1) | 6 (46.2) | 1 (25.0) | 3 (42.9) |
| No | 11 (37.9) | 7 (53.8) | 3 (75.0) | 4 (57.1) |
| ≥ 50% NRS reduction |  |  |  |  |
| Yes | 17 (58.6) | 6 (46.2) | 2 (50.0) | 1 (14.3) |
| No | 12 (41.4) | 7 (53.8) | 2 (50.0) | 6 (85.7) |
| ≥ 2-point NRS reduction |  |  |  |  |
| Yes | 21 (72.4) | 9 (69.2) | 3 (75.0) | 3 (42.9) |
| No | 8 (27.6) | 4 (30.8) | 1 (25.0) | 4 (57.1) |
| ≥ 6 PGIC |  |  |  |  |
| Yes | 19 (65.5) | 6 (46.2) | 2 (50.0) | 2 (28.6) |
| No | 10 (34.5) | 7 (53.8) | 2 (50.0) | 5 (71.4) |

NRS = Numerical Rating Scale; ODI = Oswestry Disability Index; PGIC = Patient Global Impression of Change.

Note: Values are frequency (%).

**Supplemental Table S7.** Categorical study outcomes by changes to medial and lateral branch lumbosacral radiofrequency ablation utilization at 12 months after basivertebral nerve ablation compared to baseline

|  | **Changes in LRFA Use from Baseline to 12 Months** | | | |
| --- | --- | --- | --- | --- |
| **Outcome** | **Yes (baseline) -> No**  **(*n* =10)** | **No (baseline) -> No**  **(*n* = 38)** | **No (baseline) -> Yes**  **(*n* = 2)** | **Yes (baseline) -> Yes**  **(*n* = 3)** |
| ≥ 15-point ODI reduction |  |  |  |  |
| Yes | 7 (70.0) | 12 (31.6) | 1 (50.0) | 1 (33.3) |
| No | 3 (30.0) | 26 (68.4) | 1 (50.0) | 2 (66.7) |
| ≥ 30% ODI reduction |  |  |  |  |
| Yes | 8 (80.0) | 18 (47.4) | 1 (50.0) | 1 (33.3) |
| No | 2 (20.0) | 20 (52.6) | 1 (50.0) | 2 (66.7) |
| ≥ 50% NRS reduction |  |  |  |  |
| Yes | 7 (70.0) | 18 (47.4) | 0 (0.0) | 1 (33.3) |
| No | 3 (30.0) | 20 (52.6) | 2 (100.0) | 2 (66.7) |
| ≥ 2-point NRS reduction |  |  |  |  |
| Yes | 7 (70.0) | 25 (65.8) | 2 (100.0) | 2 (66.7) |
| No | 3 (30.0) | 13 (34.2) | 0 (0.0) | 1 (33.3) |
| ≥ 6 PGIC |  |  |  |  |
| Yes | 7 (70.0) | 20 (52.6) | 1 (50.0) | 1 (33.3) |
| No | 3 (30.0) | 18 (47.4) | 1 (50.0) | 2 (66.7) |

LRFA = lumbosacral radiofrequency ablation; NRS = Numerical Rating Scale; ODI = Oswestry Disability Index; PGIC = Patient Global Impression of Change.

Note: Values are frequency (%).
